# Differences and related physiological mechanisms in effects of ammonium on the invasive plant *Xanthium strumarium* and its native congener *X. sibiricum*

**DOI:** 10.3389/fpls.2022.999748

**Published:** 2022-10-05

**Authors:** Zheng Zhang, Chang Zhang, Chun-Sha Zhang, Wei-Bin Wang, Yu-Long Feng

**Affiliations:** Liaoning Key Laboratory for Biological Invasions and Global Changes, College of Bioscience and Biotechnology, Shenyang Agricultural University, Shenyang, China

**Keywords:** ammonium, antioxidants, biological invasion, reactive oxygen metabolism, uptake, toxicity, *Xanthium strumarium*, *X. sibiricum*

## Abstract

Few studies explore the effects of nitrogen forms on exotic plant invasions, and all of them are conducted from the perspective of nitrogen form utilization without considering the effects of ammonium toxicity. The invasive plant *Xanthium strumarium* prefers to use nitrate, while its native congener *X. sibiricum* prefers to use ammonium, and the invader is more sensitive to high ammonium based on our preliminary observations. To further reveal the effects of nitrogen forms on invasiveness of *X. strumarium*, we studied the difference and related physiological mechanisms in sensitivity to ammonium between these species. With increasing ammonium, total biomass, root to shoot ratio and leaf chlorophyll content of *X. strumarium* decreased, showing ammonium toxicity. For *X. sibiricum*, however, ammonium toxicity did not occurr. With increasing ammonium, ammonium concentration increased in leaves and roots of *X. strumarium*, which is associated with the decreased activities of glutamine synthetase and glutamate synthase and the increased ammonium uptake; and consequently the contents of hydrogen peroxide and malondialdehyde also increased, which is associated with the decreased contents of reduced glutathione and ascorbic acid. By contrast, the abilities of ammonium assimilation and antioxidation of *X. sibiricum* were less affected by the increase of ammonium, and the contents of ammonium nitrogen, hydrogen peroxide and malondialdehyde in leaves and roots were significantly lower than those in *X. strumarium*. Our results indicate that ammonium accumulation and oxidative damage may be the physiological mechanisms for the ammonium toxicity of *X. strumarium*, providing a possible explanation that it generally invades nitrate-dominated and disturbed habitats and a theoretical basis for future studies on the control of invasive plants by regulating soil nitrogen.

## Introduction

Nitrogen is one of the key factors affecting plant growth, development and yield (Lea and Azevedo, 2006; Feng et al., 2009). A large number of studies have shown that atmospheric nitrogen deposition and soil nitrogen increase can promote invasions of exotic plants (Lei et al., 2012; Sun et al., 2021; Luo et al., 2022). Ammonium (NH_4_^+^) and nitrate (NO_3_^-^) are two main nitrogen forms for plants (Wang et al., 2009), and the main soil nitrogen form (Its content is higher than those of other nitrogen forms) are different in different environments. Nitrate is often the main form of soil nitrogen in disturbed habitats with severe damage from invasive plants (Sun et al., 2021; Luo et al., 2022). In general, ammonium nitrogen concentration in soil solution varies over several orders of magnitude (0.1-10 mmol L^-1^; Miller et al., 2007), and soil nitrate nitrogen content is 10-1000 times higher than that of ammonium nitrogen (Cawas and Robert, 2006). However, atmospheric ammonium nitrogen deposition, soil low temperature, waterlogging, acidification and other factors can also make soil ammonium nitrogen content reach a higher level (Li, 2013; Sun et al., 2021), forming a regional or local habitat with high NH_4_^+^. It has been shown that ammonium nitrogen concentration in farmland soil solution can increase to 2-20 mmol L^-1^ (Kronzucker et al., 2003; Han et al., 2019), decreasing crop growth and yield. However, plants are different in the sensitivity to relatively high levels of ammonium (Britto and Kronzucker, 2002; Li et al., 2020). Therefore, the difference in response to soil nitrogen forms between invasive and co-occurring native plants may affect exotic plant invasion and their habitat selection. However, there are few related references, and the existing studies mainly focus on the utilization of nitrogen forms without considering the toxic effect of ammonium nitrogen (Sun, 2017; Sun et al., 2021; Luo et al., 2022).

In many ecosystems, most plants prefer to absorb nitrate, and the increase in soil nitrate content is conducive to plant growth and exotic plant invasions (Alboresi et al., 2005; Wang et al., 2012; Luo et al., 2022). However, after absorbed by plants, nitrate has to be reduced into ammonium for further use by plants. Thus, the absorption and assimilation processes of nitrate by plants are more complex than those of ammonium, and it is more favorable for plants to use ammonium as their main nitrogen source from the perspective of energy consumption (Li and Tong, 2007). Some plants indeed prefer ammonium nitrogen and grow better under ammonium relative to nitrite (Chen et al., 2015; Luo et al., 2022). However, most plants are sensitive to ammonium, and high ammonium concentration can produce toxic effects, mainly manifested as leaf chlorosis; decreased leaf area; stubby root system; decreased root to shoot ratio, biomass and net photosynthetic rate, etc. (Britto and Kronzucker, 2002; Zou et al., 2004; Li et al., 2010; Zou et al., 2012; Jian et al., 2018). Ammonium can also lead to cytoplasmic pH disorder and carbon and nitrogen metabolism imbalance (Ou, 2018; Han et al., 2019). In addition, high ammonium levels can lead to accumulation of the superoxide radical and H_2_O_2_ in plant cells, causing oxidative damage (Liu et al., 2004).

Plants absorb ammonium from soil primarily through the ammonium transporter (AMT) in root system (Sohlenkamp et al., 2003). *In vivo*, ammonium is mainly catalyzed by glutamine synthetase (GS) and glutamate synthase (GOGAT) to produce glutamine and glutamic acid, which undergo a series of metabolic reactions to produce other organic nitrogen-containing compounds (Lea and Miflin, 2003). The differences in ammonium uptake, assimilation and the ability to regulate above processes among different plants may affect their sensitivity to ammonium. For example, rice accumulates less NH_4_^+^ under high ammonium levels compared with barley, which makes it insensitive to ammonium toxicity (Cruz et al., 2006). In addition, improving the antioxidant capacity may also alleviate ammonium toxicity for plants.

The invasive plant *Xanthium strumarium* mainly invades disturbed habitats with nitrate as the dominant nitrogen form in soil (Feng, 2020), and prefers to use nitrate, while its co-occurring native plant *X. sibiricum* prefers to use ammonium (Luo et al., 2022). Our preliminary observation has shown that *X. strumarium* shows obvious symptoms of ammonium toxicity under high ammonium levels, while *X. sibiricum* does not. In order to determine the differences and related physiological mechanisms in the sensitivity to ammonium between the two *Xanthium* species, the effects of different ammonium concentrations on growth, ammonium accumulation and assimilation, and reactive oxygen species metabolism of these species were explored in this study. We hypothesize that compared with *X. sibiricum*, (1) *X. strumarium* may accumulate more ammonium in roots and leaves under high ammonium levels due to its low ammonium assimilation ability; (2) the accumulated ammonium may decrease its antioxidant capacity, resulting in the accumulation of more reactive oxygen species and thus oxidative damage. Our study will contribute to understanding not only the reasons that *X. strumarium* mainly invades nitrate-dominant habitats and the impacts of soil nitrogen forms on exotic plant invasions from the perspective of ammonium stress, but also the physiological mechanisms underlying the difference in ammonium sensitivity among different plants.

## Materials and methods

### Study materials

In this study, the invasive plant *Xanthium strumarium* and its co-occurring native congener *X. sibiricum* were used as research materials (**Figure S1**). *Xanthium strumarium*, an annual herb of Asteraceae, is originated from North America, and now widely distributed in abandoned lands, pastures, riparian beaches, humid grasslands and other habitats in northeast China, north China, and northwest China (Feng, 2020). *Xanthium sibiricum* is native to China, and widely distributed in China, mainly in the plains, hills, low mountains, wilderness, and grassland beside fields.

The seeds of the two *Xanthium* specie were collected in Hunnan District of Shenyang City, Liaoning Province in the autumn of 2019, and stored at 4 °C after dried in the room. To improve seed germination, low temperature stratification treatment was conducted from winter of 2019 to spring of 2020.

### Experiment design

In this study, two experiments were carried out. The first one was to explore the effects of ammonium with different concentrations on the two *Xanthium* species and the interspecific differences, identifying the toxic effects and toxic concentration of ammonium on these species. The second one was to explore the physiological mechanisms underlying (alleviating) ammonium toxicity for the two species.

#### Experiment 1

##### Seeds germination

The healthy and similar-sized fruits of *X. strumarium* and *X. sibiricum* were selected, the peel was removed, and the superior seed was collected for each fruit. The seeds were disinfected with 3% NaClO solution for 15 min, rinsed with distilled water for 4 times, put into petri dishes with wet filter papers, and then placed in HPG-400HX growth chamber (Harbin Donglian technology Co., Ltd, Harbin, China) for germination. In the chamber, light intensity was 32 μmol m^-2^ s^-1^ with photoperiod of 12 h/12 h, and day/night temperature 28°C/25°C, and humidity 50%. During germination, distilled water was added into each petri dish every day to ensure appropriate water status for seeds germination.

##### Seedling transplantation

After one week of seed germination, ≈5 cm tall seedlings of the two *Xanthium* species were selected, transplanted into pots (7 × 7 × 10 cm, one per pot) filled with vermiculite, and placed in an growth room for culture. In the room, light intensity was 200 μmol m^-2^ s^-1^ with photoperiod of 16 h/8 h, and temperature 28 °C. The seedlings were watered with modified Hoagland nutrient solution (100 ml, pH 5.5) every two days. The nutrient solution contained 1652 mg L^-1^ Ca(NO_3_)_2_·4H_2_O, 66 mg L^-1^ (NH_4_)_2_SO_4_, 493 mg L^-1^ MgSO_4_·7H_2_O, 136 mg L^-1^ KH_2_PO_4_, 372 mg L^-1^ KCl, 27.8 mg L^-1^ FeSO_4_·7H_2_O, 12 mg L^-1^ Na_2_EDTA, 2.86 mg L^-1^ H_3_BO_3_, 2.13 mg L^-1^ MnSO_4_·H_2_O, 0.22 mg L^-1^ ZnSO_4_·7H_2_O, 0.08 mg L^-1^ CuSO_4_·5H_2_O, 0.02 mg L^-1^ (NH_4_)_6_Mo_7_O_24_·4H_2_O. All of the reagents used were analytically pure.

##### Ammonium treatmen

When the seedlings were ≈10 cm high and had two pairs of leaves per seedling, similar-sized seedlings were selected and treated with eight concentrations of ammonium (0.25, 0.5, 1.0, 2.5, 5.0, 7.5, 10.0 and 12.5 mmol L^-1^), 10 pots per treatment. These concentrations of ammonium nitrogen was used according to our preliminary experiments (including the concentrations with toxic effects), which were within the range of ammonium concentration of soil solutions in the field (Kronzucker et al., 2003; Miller et al., 2007; Han et al., 2019). The Hoagland nutrient solution contained 0.5 mmol L^-1^ nitrate as the seedlings of the two *Xanthium* species grew badly without nitrate, and CaCl_2_ was used to replace the excess Ca(NO_3_)_2_·4H_2_O in the nutrient solution. The contents of other elements were not changed in the solution. 7 μmol L^-1^ C_2_H_2_N_4_ was added into the solution to inhibit nitrification. The seedlings were watered with the nutrient solution every two days.

##### Sampling measurement

After treated with different concentrations of ammonium for 7 d, appearance features such as leaf shape, color and size were observed. Six seedlings (6 replicates) were randomly selected for each species and treatment, and leaf chlorophyll content, total biomass and root to shoot ratio were measured (See measurement section).

#### Experiment 2

According to the effects of ammonium on the two *Xanthium* species in experiment 1, three ammonium concentrations were used in this experiment (15 pots per treatment): 8.0 mmol L^-1^ (with significantly toxic effects, 2.0 mmol L^-1^ (with lightly or no toxic effects) and 0.5 mmol L^-1^ (with non-toxic effects). The methods of seed germination, seedling transplantation and ammonium treatment were the same as those in experiment 1. After treated with different concentrations of ammonium for 7 d, appearance features such as leaf shape, color and size were observed, and leaf chlorophyll content was measured. Twelve seedlings (2 as a replicate as the leaves and fine roots of one seedling were not enough for measurements) were randomly selected for each species and treatment, and recently matured leaves and fine roots were collected, and divided into two parts, respectively. One part of each organ was dried at 60 °C for determination of the total nitrogen concentration, and one part was quickly frozen in liquid nitrogen for 15 min, and stored in -80 °C DW-86L388A refrigerator (Haier group, Qingdao, China) for determination of the ammonium nitrogen concentration, the activities of glutamine synthetase and glutamate synthase, and the contents of malondialdehyde, hydrogen peroxide (H_2_O_2_), reduced glutathione and ascorbic acid, all with 6 replicates.

### Measurements

#### Leaf relative chlorophyll content

Determination was performed using SPAD-502 Plus chlorophyll meter (Konica Minolta, Tokyo, Japan).

#### Total biomass and root to shoot ratio

The shoots and roots of the seedlings were collected separately for each species, washed with water, dried with filter paper, dried at 60°C to constant weight in GZX-9246 MBE oven (Bosun, Shanghai, China), and weighed separately with Sartorius BSA224S analytical balance (Shanghai Renhe Scientific Instrument, Shanghai, China). Total biomass and root to shoot ratio (root dry weight/shoot dry weight) were calculated for each seedling.

#### Leaf and root ammonium nitrogen concentrations

The determination was performed using the ammonium nitrogen kit (Keming Biotechnology, Suzhou, China). Under strong alkaline condition, ammonium was reacted with phenol and hypochlorite to produce indophenol blue, and the absorbance at 625 nm was measured to obtain the ammonium nitrogen concentration.

#### Leaf and root total nitrogen concentrations

The samples were separately ground into powder using the GT200 vibration grinder (Beijing Grinder Instrument Equipment, Beijing, China), and 0.3 g powder was used to determine total nitrogen content using the EA3000 elemental analyzer (Euro Vector, Milan, Italy).

#### Leaf and root ammonium assimilation enzyme activities

The activities of glutamine synthetase (GS; EC 6. 3. 1. 2) and glutamate synthase (GOGAT; EC 1. 4. 1. 14) were measured using the GS kit and the GOGAT kit (Keming Biotechnology, Suzhou, China), respectively. GS catalyzed the synthesis of glutamine by NH_4_^+^ and glutamic acid in the presence of Mg^2+^ and ATP. The glutamine was further reacted with iron to form a complex in acidic medium, and the absorbance of the reaction solution was measured at 540 nm to calculate GS activity. GOGAT catalyzed the amino group of glutamine to transfer to α-ketoglutaric acid to form glutamic acid, and NADH was oxidized to NAD^+^, and the decreasing rate of absorbance at 340 nm was measured to calculate the GOGAT activity.

#### Leaf and root malondialdehyde contents

The determination was performed using the malondialdehyde content kit (Keming Biotechnology, Suzhou, China). Malondialdehyde and thiobarbituric acid (TBA) was condensed to produce a red product, and the absorbance of the reaction solution was measured at 532 nm to estimate the content of lipid peroxide. Meanwhile, the absorbance at 600 nm was measured, and the malondialdehyde content was calculated by the difference in the absorbance at 532 nm and 600 nm.

#### Leaf and root H_2_O_2_ contents

The H_2_O_2_ content was measured using the H_2_O_2_ kit (Keming Biotechnology, Suzhou, China). H_2_O_2_ was reacted with Ti(SO_4_)_2_ to generate a yellow titanium peroxide compound, and the absorbance was measured at 415 nm to calculate the H_2_O_2_ content.

#### Leaf and root antioxygen contents

The contents of reduced glutathione (GSH) and ascorbic acid (AsA) were measured using the GSH kit and AsA kit (Keming Biotechnology, Suzhou, China), respectively. GSH was reacted with DTNB, and the absorbance of the reaction solution was measured at 412 nm to calculate the GSH content. AsA was reacted with fast blue salt B under acidic conditions to generate yellow oxalohydrazine-2-hydroxyl butylinolide derivatives, and the absorbance was measured at 420 nm to calculate AsA content.

Sample pretreatment and determination were performed according to the instructions of the kits, and the absorbances were determined using the Multiskan GO microplate readers (ThermoFisher, Massachusetts, USA).

### Data analysis

Two-way analysis of variance (ANOVA) was used to test the effects of species, ammonium concentration and their interaction on each parameter. One-way ANOVA was used to test the difference in each parameter between different ammonium treatments in *X. strumarium* and *X. sibiricum*, respectively. Independent sample *t*-test was used to analyze the interspecific differences under the same ammonium treatment. Before statistical analysis, the homogeneity of variance and normal distribution of each parameter were tested, and the ANOVA conditions were met for all parameters. SPSS 25.0 (SPSS Inc., Chicago, IL, USA) software was used for all statistical analyses. Sigmaplot 14.0 (systat software Inc., San Jose, CA, USA) software was used for drawing figures.

## Results

### Total biomass, root to shoot ratio and leaf chlorophyll contents

**Figure 1:**
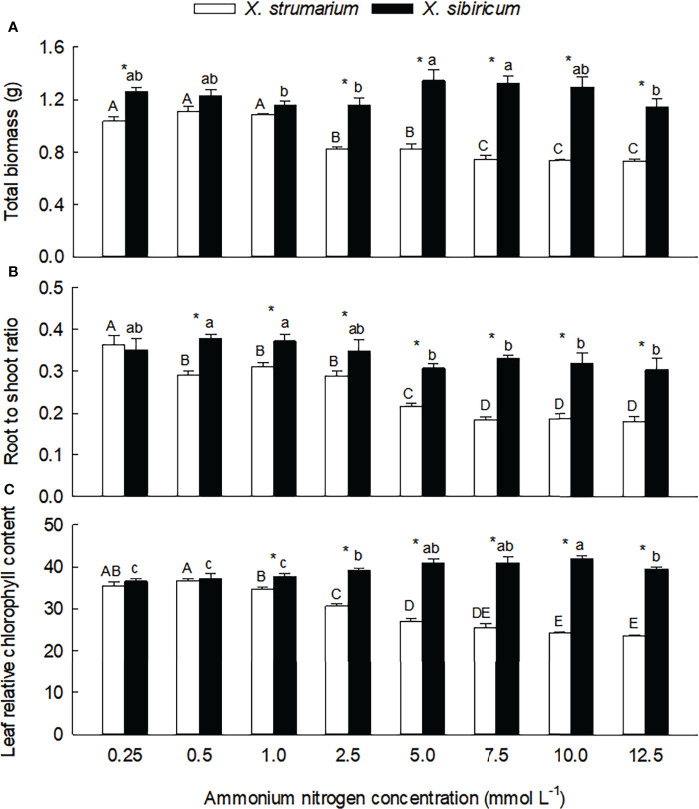
Differences in total biomass **(A)**, root to shoot ratio **(B)** and leaf relative chlorophyll content **(C)** between the invasive plant *Xanthium strumarium* and its native congener *X. sibiricum* under different ammonium levels. Means + 1 SE (*n* = 6). Different upper- and lowercase letters indicate significant differences among ammonium treatments for *X. strumarium* and *X. sibiricum*, respectively (*P* < 0.05; one-way ANOVA); * indicates significant difference between the two species under the same ammonium treatment (*P* < 0.05; independent sample *t*-test). See **Table S1** for the results of two-way ANOVA.

Species, ammonium concentration and their interaction all significantly influenced total biomass, root to shoot ratio and leaf relative chlorophyll content (**Table S1**), indicating that the invasive plant *Xanthium strumarium* and its native congener *X. sibiricum* responded differently to ammonium concentration. Under low ammonium levels, the interspecific differences in total biomass (0.5 and 1.0 mmol L^-1^), root to shoot ratio (0.25 mmol L^-1^) and leaf chlorophyll content (0.25 and 0.5 mmol L^-1^) were not significant (**Figure 1**). Under high ammonium levels, these traits were all significantly lower in *X. strumarium* compared with *X. sibiricum*.

Under low ammonium levels (0.25 - 1.0 mmol L^-1^), total biomass did not changed significantly in both of the *Xanthium* species (**Figure 1A**). When the ammonium concentration increased to 2.5 mmol L^-1^, total biomass of *X. strumarium* decreased significantly, then decreased gradually with the increase of ammonium concentration, and reached the lowest value under the concentration of 10.0 mmol L^-1^. For *X. sibiricum*, however, total biomass did not decrease significantly with increasing ammonium concentrations.

Root to shoot ratio of *X. strumarium* was more sensitive to ammonium concentration than total biomass (**Figure 1B**): more early decreased (under 0.5 vs. 2.5 mmol L^-1^) and reached the lowest value (under 7.5 vs. 10.0 mmol L^-1^). For *X. sibiricum*, root to shoot ratio did not change significantly under 0.5 - 2.5 mmol L^-1^ ammonium concentrations. Under 5 and 12.5 mmol L^-1^ ammonium concentrations, the values of root to shoot ratio were significantly lower than those under 0.5 and 1.0 mmol L^-1^ ammonium concentrations, but similar with those under 0.25 and 2.5 mmol L^-1^ ammonium concentrations. Under 7.5 and 10.0 mmol L^-1^ ammonium concentrations, however, the values of root to shoot ratio were not significantly different with those under other ammonium concentrations.

Under low ammonium levels (0.25 and 0.5 mmol L^-1^), the changes of leaf chlorophyll contents were not significant for both of the *Xanthium* species (**Figure 1C**). With the increase of ammonium concentration, leaf chlorophyll content of *X. strumarium* decreased gradually, and reached the lowest value under 7.5 mmol L^-1^ ammonium, while increased gradually in *X. sibiricum*. As for the effects of ammonium concentration on leaf chlorophyll content and its interspecific difference, similar results were also found in our second experiment (**Figure S2**).

Under high ammonium, the leaves of *X. strumarium* lost green seriously, with slightly curled edge, dry top and slender shape, and growth were severely inhibited (**Figures S3** and **S4**). In contrast, the appearance features such as leaves shape, size, color of *X. sibiricum* were not significantly changed under different ammonium concentrations.

### Ammonium and total nitrogen contents and ammonium assimilation enzyme activities

**Figure 2:**
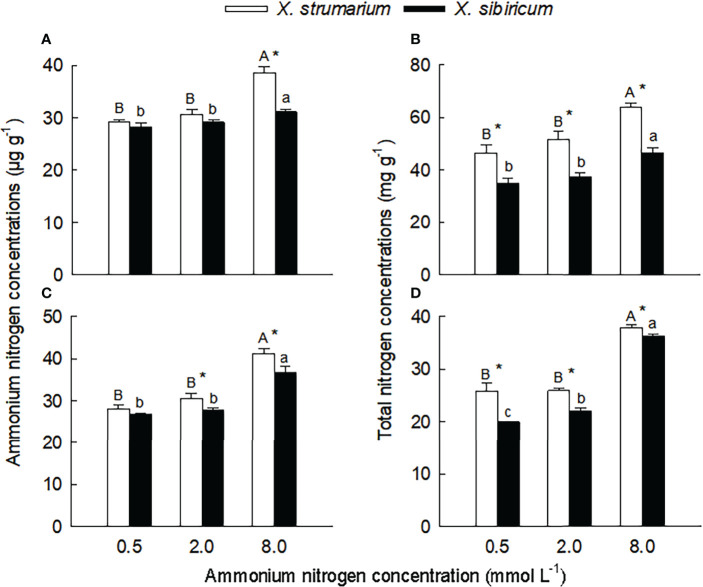
Differences in leaf **(A, B)** and fine root **(C, D)** concentrations of ammonium **(A, C)** and total **(B, D)** nitrogen between the invasive plant *Xanthium strumarium* and its native congener *X. sibiricum* under different ammonium levels. Means + 1 SE (*n* = 6). Different upper- and lowercase letters indicate significant differences among ammonium treatments for *X. strumarium* and *X. sibiricum*, respectively (*P* < 0.05; one-way ANOVA); * indicates significant difference between the two species under the same ammonium treatment (*P* < 0.05; independent sample *t*-test). See **Table S2** for the results of two-way ANOVA.

Species, ammonium concentrations and their interaction all significantly influenced ammonium and total nitrogen (except the interaction for fine roots) concentrations, glutamine synthetase (except ammonium concentrations for leaves) and glutamate synthase activities in leaves and fine roots (**Table S2**), indicating that *X. strumarium* and *X. sibiricum* responded differently to ammonium concentration. Under the low ammonium (0.5 mmol L^-1^), the interspecific differences in leaf and fine root ammonium nitrogen concentration were not significant (**Figures 2A, C**). Under the intermediate (0.2 mmol L^-1^) and high (8.0 mmol L^-1^) ammonium levels, the ammonium nitrogen concentrations in leaves (not significant under 0.2 mmol L^-1^ ammonium) and fine roots were significantly higher for *X. strumarium* than for *X. sibiricum*. The magnitude of the interspecific difference increased with increasing ammonium levels. Under all three ammonium treatments, leaf and root total nitrogen content was significantly higher for *X. strumarium* than for *X. sibiricum* (**Figures 2B, D**).

Under the low and intermediate ammonium levels, leaf and root concentrations of ammonium and total nitrogen did not significantly changed for both species (except for total nitrogen concentration in fine roots of *X. sibiricum*) (**Figure 2**). When the ammonium concentration increased to 8.0 mmol L^-1^, leaf and root concentrations of ammonium and total nitrogen significantly increased, and the increase was more significant in *X. strumarium* relative to *X. sibiricum*.

**Figure 3:**
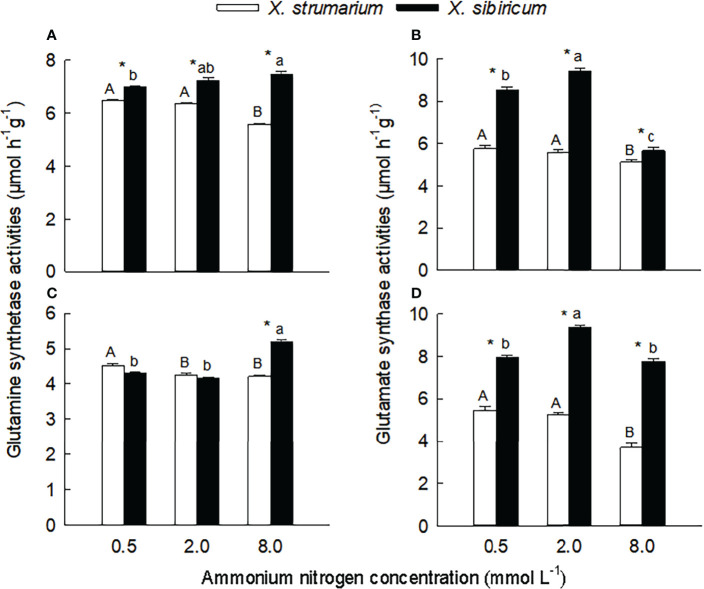
Differences in leaf **(A, B)** and fine root **(C, D)** activities of glutamine synthetase **(A, C)** and glutamate synthase **(B, D)** between the invasive plant *Xanthium strumarium* and its native congener *X. sibiricum* under different ammonium levels. Means + 1 SE (*n* = 6). Different upper- and lowercase letters indicate significant differences among ammonium treatments for *X. strumarium* and *X. sibiricum*, respectively (*P* < 0.05; one-way ANOVA); * indicates significant difference between the two species under the same ammonium treatment (*P* < 0.05; independent sample *t*-test). See **Table S2** for the result of two-way ANOVA.

Under each ammonium treatment, the activities of glutamine synthetase in leaves, and glutamate synthase in leaves and fine roots were significantly lower for *X. strumarium* than for *X. sibiricum* (**Figures 3A, B, D**). Under high ammonium level, the activity of glutamine synthetase in fine roots of *X. strumarium* was significantly lower than that of *X. sibiricum*, and the interspecific difference was not significant under low and intermediate ammonium levels.

With the increase of ammonium levels, the activities of glutamine synthetase and glutamate synthase showed decreasing trends in leaves and fine roots of *X. strumarium* (**Figure 3**). In leaves and fine roots of *X. sibiricum*, however, with increasing ammonium concentration the glutamine synthetase activities significantly increased, and the glutamate synthase activities increased first and then decreased.

### Malondialdehyde, H_2_O_2_ and antioxidant contents

**Figure 4:**
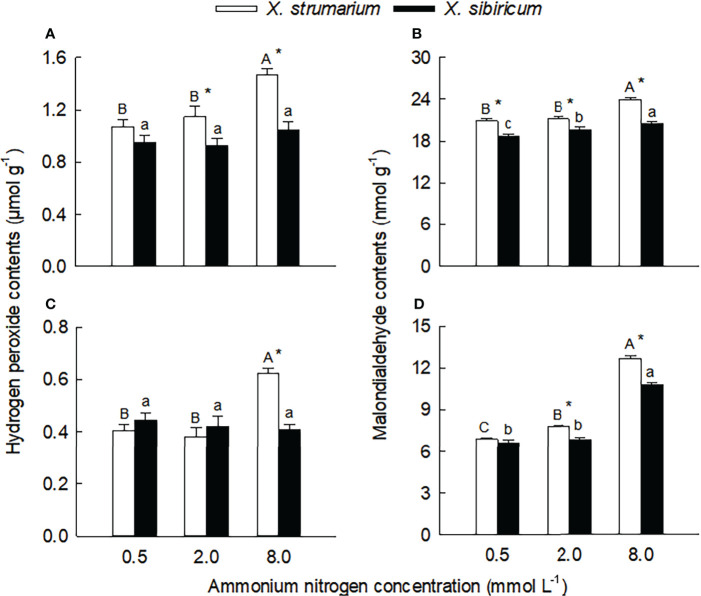
Differences in leaf **(A, B)** and fine root **(C, D)** contents of malondialdehyde **(A, C)** and hydrogen peroxide **(B, D)** between the invasive plant *Xanthium strumarium* and its native congener *X. sibiricum* under different ammonium levels. Means + 1 SE (*n* = 6). Different upper- and lowercase letters indicate significant differences among ammonium treatments for *X. strumarium* and *X. sibiricum*, respectively (*P* < 0.05; one-way ANOVA); * indicates significant difference between the two species under the same ammonium treatment (*P* < 0.05; independent sample *t*-test). See **Table S2** for the results of two-way ANOVA.

Species, ammonium concentration and their interaction all significantly influenced leaf and root contents of H_2_O_2_, malondialdehyde, and reduced glutathione (except ammonium concentration in leaves) and ascorbic acid (except ammonium concentration in fine roots) (**Table S2**), indicating that the two *Xanthium* species responded differently to ammonium concentration. Under intermediate and high ammonium levels, the H_2_O_2_ contents in leaves of *X. strumarium* was higher than those of *X. sibiricum*, and under high ammonium concentrations, the H_2_O_2_ content in fine roots of *X. strumarium* was higher than that of *X. sibiricum* (**Figures 4A, C**). Under high ammonium concentrations, the H_2_O_2_ contents in leaves and fine roots of *X. strumarium* increased significantly, while the changes in *X. sibiricum* were not significant.

**Figure 5:**
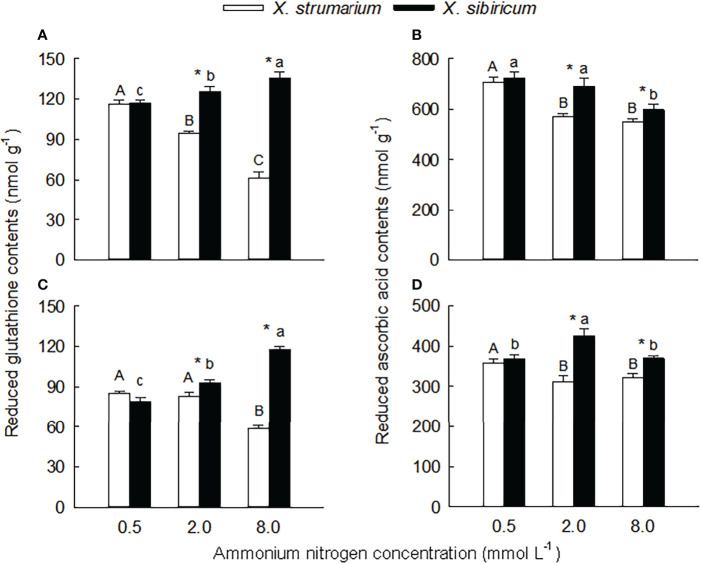
Differences in leaf **(A, B)** and fine root **(C, D)** contents of reduced glutathione **(A, C)** and ascorbic acid **(B, D)** between the invasive plant *Xanthium strumarium* and its native congener *X. sibiricum* under different ammonium levels. Means + 1 SE (*n* = 6). Different upper- and lowercase letters indicate significant differences among ammonium treatments for *X. strumarium* and *X. sibiricum*, respectively (*P* < 0.05; one-way ANOVA); * indicates significant difference between the two species under the same ammonium treatment (*P* < 0.05; independent sample *t*-test). See **Table S2** for the results of two-way ANOVA.

Under each ammonium treatment, the malondialdehyde contents in leaves and fine roots of *X. strumarium* were significantly higher than those of *X. sibiricum* (except for root under low ammonium) (**Figures 5B, D**). With the increase of ammonium concentration, the malondialdehyde contents in leaves and fine roots of the two *Xanthium* species increased significantly, and the magnitude of the increase was greater for *X. strumarium*.

Under the intermediate and high ammonium levels, the contents of reduced glutathione and ascorbic acid in leaves and fine roots of *X. strumarium* were significantly higher than those of *X. sibiricum*, while the interspecific difference was not significant under the low ammonium concentration (**Figure 5**).

With the increase of ammonium levels, the contents of reduced glutathione and ascorbic acid in leaves and fine roots of *X. strumarium* and the reduced ascorbic acid content in leaves of *X. sibiricum* showed decreasing trends (**Figure 5**). However, in leaves and fine roots of *X. sibiricum*, the reduced glutathione contents increased significantly, and the reduced ascorbic acid contents increased first and then decreased.

## Discussion

Our study showed that the invasive plant *Xanthium strumarium* was more sensitive to ammonium than its native congener *X. sibiricum*. In leaves and fine roots of the invader, the activities of ammonium assimilation enzymes decreased and the contents of ammonium nitrogen and H_2_O_2_ increased with increasing ammonium levels, causing oxidative damage. These results may explain the reason why *X. strumarium* prefers to invade the habitats with nitrate as the main nitrogen form in soil from a new perspective. The previous study conducted by our laboratory has shown that *X. strumarium* prefers to absorb nitrate relative to ammonium and has higher plasticity to nitrate (Luo et al., 2022), which is considered to be the important reason for the invader to invade nitrate-dominated soils. Some studies have shown the effects of nitrogen forms on exotic plant invasions from the perspective of nitrogen form utilization (Sun et al., 2021). However, no effort has been made to study the effects of nitrogen forms from the perspective of ammonium toxicity.

## Effects of ammonium on growth

Consistent with our hypothesis, *X. strumarium* was more sensitive to ammonium than *X. sibiricum*. Under high ammonium, the invader showed obvious symptoms of ammonium toxicity, while *X. sibiricum* did not. When ammonium level increased to 2.5 mmol L^-1^, total biomass and leaf chlorophyll content decreased significantly in *X. strumarium*, and its leaf color and morphology also changed significantly. The higher the ammonium level was, the greater the harmful effects were. Chlorophyll is closely associated with plant photosynthesis. Claussen and Lenz (1999) also found that chlorophyll content of cucumber decreases under high ammonium. With increasing ammonium levels, leaf and fine root malondialdehyde contents increased more greatly in the invader compared with *X. sibiricum*, indicating that oxidative stress occurred and membrane lipid peroxidation aggravated (Jin et al., 2008), which may be the important reason for the decreased leaf chlorophyll content and total biomass. Studies on ammonium toxicity have mainly focused on crops (Liu et al., 2004; Liu, 2017; Jian et al., 2018), and no study has been conducted using invasive and native plants.

Compared with total biomass, root to shoot ratio of *X. strumarium* was more sensitive to ammonium, which began to decrease under a lower ammonium level. Root system directly contacts with soil ammonium and is also the organ for ammonium to enter and accumulate first, which may explain the reasons why root was more sensitive to ammonium than shoot. Britto and Kronzucker (2002) and Liu (2017) also found that root system is more sensitive to high ammonium than shoot. Growth of seed root and lateral root of rice seedlings is significantly inhibited under high ammonium (Hirano et al., 2013).

Our study showed that different plants and different organs of the same plant had different sensitivity to ammonium, but the physiological mechanisms underlying these differences are still not clear. Consistently, leaf chlorophyll content of cucumber decreases significantly under high ammonium, while that of rice does not (Claussen and Lenz, 1999). Under high ammonium, shoot and root dry weights are always higher in ammonium-tolerant wheat variety than ammonium-sensitive wheat variety (Li, 2013).

## Ammonium accumulation

Consistent with our hypothesis, the ammonium nitrogen concentrations in leaves and fine roots of the two *Xanthium* species increased significantly with increasing ammonium levels, and magnitude of the increase was greater in *X. strumarium*. The accumulation of ammonium in leaves and roots may be at least one of the reasons for ammonium toxicity in *X. strumarium*. High ammonium not only affects growth of most plants, but also affects their uptake, accumulation and distribution of nitrogen (Sun et al., 2003). Under high ammonium level, ammonium uptake rates increase in citrange seedlings, resulting in ammonium accumulation and toxic effects (Sun, 2017). Compared with barley, rice accumulates less ammonium under high ammonium treatment, and shows stronger ammonium tolerance (Cruz et al., 2006).

With increasing ammonium level, total nitrogen concentrations in leaves and fine roots increased for the two *Xanthium* species, and were always higher for *X. strumrium* than for *X. sibiricum*. These results indicate that *X. strumarium* increased ammonium uptake more greatly than *X. sibiricum* with increasing ammonium. In contrast, the activities of glutamine synthetase and glutamate synthase in leaves and fine roots of *X. strumrium* decreased with increasing ammonium level, and were always lower than those of *X. sibiricum*. The more greatly increased uptake and decreased assimilation of ammonium may synergistically cause ammonium accumulation in leaves and roots of *X. strumrium* under high ammonium level, while *X. sibiricum* can efficiently maintain tissue ammonium at relatively low levels. Usually, ammonium absorbed by plant roots is firstly assimilated into amino acids in roots, and then transported to the xylem and shoot through specific transporters (Tegeder, 2014). If the ammonium can not be assimilated into organic nitrogen in time and accumulate in roots, ammonium toxicity may occurr. Plant ammonium contents depend on its absorption, transportation and assimilation (Tabuchi et al., 2007). Li (2013) found that ammonium-tolerant wheat variety has lower uptake and higher assimilation rates of ammonium under high level than ammonium-sensitive wheat variety.

## Oxidative damage

Consistent with our hypothesis, the contents of H_2_O_2_ and malondialdehyde in leaves and fine roots of *X. strumarium* increased significantly with increasing ammonium level, causing oxidative damage. For *X. sibiricum*, however, the effects of ammonium were less, especially on H_2_O_2_. Polesskaya et al. (2004) also found that the contents of H_2_O_2_ and malondialdehyde increased significantly in wheat under high ammonium. The decreased contents of the antioxidants (reduced glutathione and ascorbic acid) in leaves and fine roots of *X. strumarium* under high ammonium may be one of the reasons for its oxidative damage. By contrast, the increased contents of the antioxidants (especially reduced glutathione) in leaves and fine roots of *X. sibiricum* under high ammonium could help it to effectively remove reactive oxygen species, increasing its ammonium tolerance. Other plants such as apricot (Wang et al., 2013) and cabbage (Shan et al., 2018) can also alleviate oxidative damage and maintain redox balance by regulating the contents of antioxidants in the AsA-GSH cycle. In addition, antioxidant enzymes are also involved in active oxygen scavenging and enhance plant ability to resist stress (Pignocchi et al., 2006; Tewari et al., 2007). Liu (2017) found that catalase activity in roots of wheat increases under high ammonium, and the magnitude of the increase is greater in ammonium-tolerant variety than in ammonium-sensitive variety. However, we did not find consistently higher activities of antioxidant enzymes for *X. sibiricum* than for *X. stumarium* (data not shown).

From the perspective of energy consumption, using ammonium relative to nitrate is more beneficial to plants. However, most plants are sensitive to high ammonium, which may cause toxic effects. It has important theoretical and practical significance to study the physiological mechanisms of ammonium toxicity and tolerance. However, the mechanisms of ammonium toxicity are very complex, which may also be associated with ion imbalance (Szczerba et al., 2008), carbon and nitrogen metabolism imbalance (Hachiya et al., 2012) and cell acidification (Hachiya et al., 2021), besides with ammonium accumulation and active oxygen metabolism disorders. In addition, the inhibitory effect of ammonium toxicity on plant growth mainly occurs in the seedling stage of crops (Liu, 2017). At present, the physiological mechanisms of ammonium toxicity and detoxification are still unclear, and its effects on exotic plant invasions needs further investigation. Our results revealed the physiological mechanisms underlying the difference in sensitivity to ammonium between the two *Xanthim* species, and gave a novel explanation that *X. strumarium* mainly invades nitrate-dominated habitats in the novel perspective of ammonium toxicity. These results indicate that increasing soil ammonium content may inhibit invasion success of *X. strumarium*, providing a theoretical basis for future studies on prevention and control of *X. strumarium* by regulating soil nitrogen forms.

Our study was carried out using seedlings of the two *Xanthium* species, and the seedlings were treated with different concentrations of ammonium for only 7 d. We can not simply extrapolate the long-term effects of ammonium on plant growth and reproduction based on the results of our study. In addition, the conditions under which the seedlings were grown and treated with different concentrations of ammonium were different with those in the field. Thus, more studies are needed in order to comprehensively understand the effects of ammonum on invasiveness of *X. strumarium*.

## Conclusion

**Figure 6:**
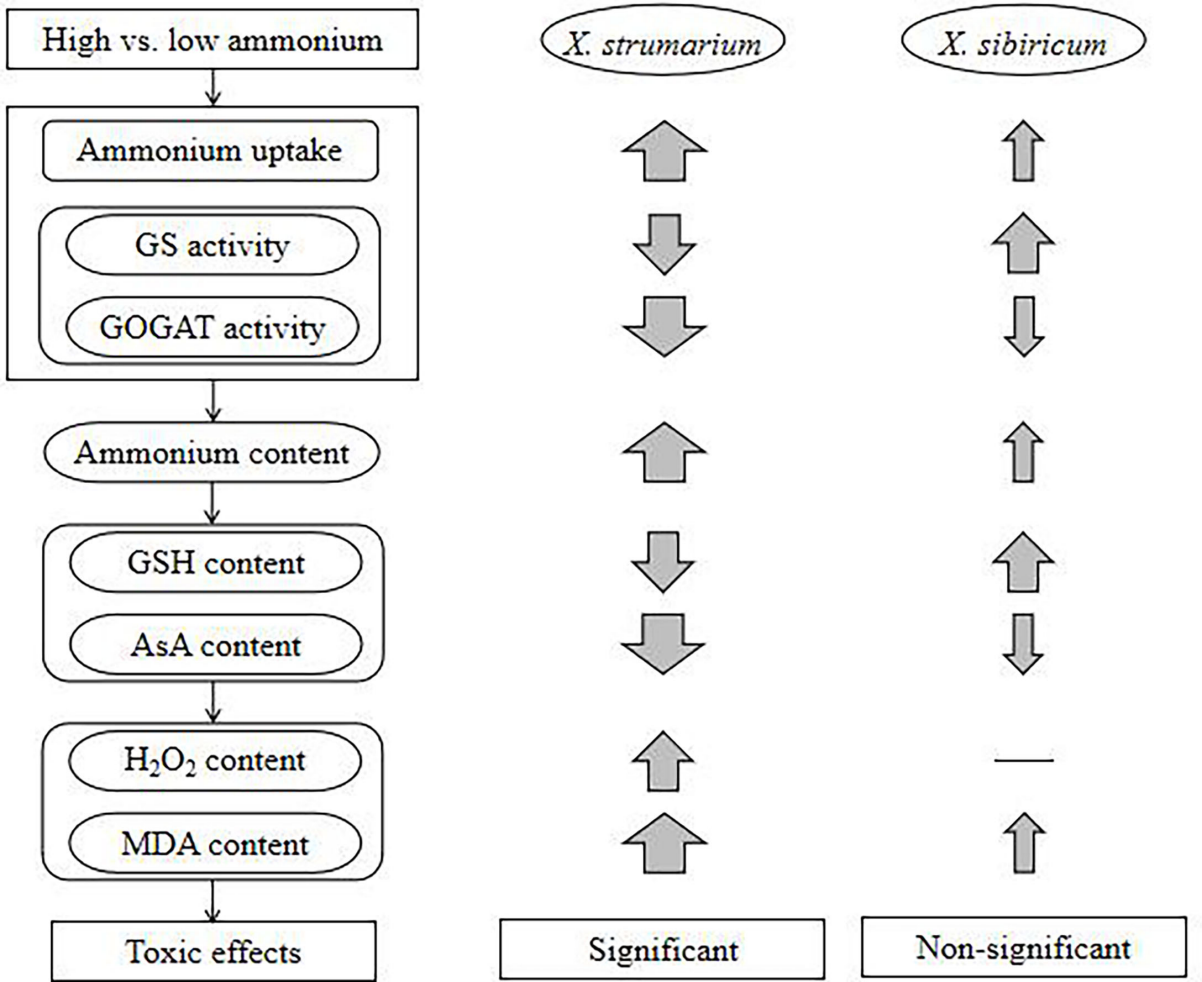
A schematic diagram showing the effects of high ammonium concentration on the invasive plant *Xanthium strumarium* and its native congener *X. sibiricum*. GS, glutamine synthetase; GOGAT, glutamate synthase; GSH, reduced glutathione; AsA, reduced ascorbic acid; H_2_O_2_, hydrogen peroxide; MDA, malondialdehyde. Up arrows, increase; down arrows, decrease; thickness of the arrow indicates the degree of increase or decrease; —, not change.

With increasing ammonium, the invasive plant *X. strumarium* increases ammonium uptake, but decreases ammonium assimilation, resulting in ammonium accumulation in leaves and fine roots, which may further lead to active oxygen metabolic disorders and oxidative stress, and membrane lipid peroxidation, reducing leaf area and chlorophyll content and thus growth (**Figure 6**). By contrast, under high ammonium the native plant *X. sibiricum* can improve ammonium assimilation and antioxidant capacity in leaves and roots, and maintain relatively low levels of ammonium and H_2_O_2_, avoiding ammonium toxicity. Our study is beneficial for understanding not only the physiological mechanism underlying the difference in ammonium toxicity among different plants, but also the effccts of soil nitrogen forms on exotic plant invasions and the selection of invasive habitats from a novel perspective of ammonium toxicity, providing a preliminary theoretical basis for controlling invasive plants by regulating soil nitrogen forms in the future.

## Data availability statement

The original contributions presented in the study are included in the article/**Supplementary Material**. Further inquiries can be directed to the corresponding author.

## Author contributions

Y-LF: conceptualization, supervision, data analysis, and writing of manuscript. ZZ: performing experiments, data analysis, and writing of manuscript. C-SZ: performing some of the experiments. CZ and W-BW: chemical analysis advice, review and editing of manuscript. All authors contributed to the article and approved the submitted version.

## Funding

This work was supported by the National Natural Science Foundation of China (31971557, 31670545 and 32001235), and the National Key Research and Development Program of China (2017YFC-1200101).

## Acknowledgments

The authors are grateful to the Analysis and Testing Center of Shenyang Agricultural University for assistance of chemical measurements, the handling editor and the anonymous reviewers for their valuable comments and suggestions on an early version of this paper.

## Conflict of interest

The authors declare that the research was conducted in the absence of any commercial or financial relationships that could be construed as a potential conflict of interest.

## Publisher’s note

All claims expressed in this article are solely those of the authors and do not necessarily represent those of their affiliated organizations, or those of the publisher, the editors and the reviewers. Any product that may be evaluated in this article, or claim that may be made by its manufacturer, is not guaranteed or endorsed by the publisher.

## References

[B1] Alboresi A. Gestin C. Leydecker M. T. Bedu M. Meyer C. Truong H. N. (2005). Nitrate, a signal relieving seed dormancy in *Arabidopsis* . Plant Cell Environ. 28, 500–512. doi: 10.1111/j.1365-3040.2005.01292.x 16229082

[B2] Britto D. T. Kronzucker H. J. (2002). NH_4_ ^+^ toxicity in higher plants: a critical review. Plant Physiol. 159, 567–584. doi: 10.1078/0176-1617-0774

[B3] Cawas E. B. Robert K. G. (2006). Reciprocal leaf and root expression of *AtAmt1.1* and root architectural changes in response to nitrogen starvation. Plant Physiol. 143, 236–250. doi: 10.1104/pp.106.088500 17085512 PMC1761975

[B4] Chen J. Y. Gu Y. R. Tian X. S. Li W. H. (2015). Responses of the invasive plant wedelia trilobata to NH_4_ ^+^-n and NO_3_ ^–^N. J. South Chin. Norm. Univ. (Nat. Sci. Ed.) 47, 84–90. doi: 10.6054/j.jscnun.2015.04.003

[B5] Claussen W. Lenz F. (1999). Effect of ammonium or nitrate nutrition on net photosynthesis, growth, and activity of the enzymes nitrate reductase and glutamine synthetase in blueberry, raspberry and strawberry. Plant Soil 208, 95–102. doi: 10.1023/A:1004543128899

[B6] Cruz C. Bio A. F. M. Domínguez-Valdivia M. D. Aparicio-Tejo P. M. Lamsfus C. Martins-Loução M. A. (2006). How does glutamine synthetase activity determine plant tolerance to ammonium? Planta 223, 1068–1080. doi: 10.1007/s00425-005-0155-2 16292661

[B7] Feng Y.-L. (2020). Invasive plants in northeast China (Beijing: Science Publication House).

[B8] Feng Y.-L. Lei Y.-B. Wang R.-F. Callaway R.-M. Valiente-Banuet A. Inderjit A.. (2009). Evolutionary tradeoffs for nitrogen allocation to photosynthesis versus cell walls in an invasive plant. Proc. Nat. Acad. Sci. U.S.A. 106, 1853–1856. doi: 10.1073/pnas.0808434106 PMC2644127 19171910

[B9] Hachiya T. Inaba J. Wakazaki M. Sato M. Toyooka K. Miyagi A.. (2021). Excessive ammonium assimilation by plastidic glutamine synthetase causes ammonium toxicity in *Arabidopsis thaliana* . Nat. Commun. 12, 4944–4953. doi: 10.1038/s41467-021-25238-7 34400629 PMC8367978

[B10] Hachiya T. Watanabe C. K. Fujimoto M. Ishikawa T. Takahara K. Kawai-Yamada M.. (2012). Nitrate addition alleviates ammonium toxicity without lessening ammonium accumulation, organic acid depletion and inorganic cation depletion in *Arabidopsis thaliana* shoots. Plant Cell Physiol. 53, 577–591. doi: 10.1093/pcp/pcs012 22318863

[B11] Han Q. F. Chen H. F. Zhang Z. H. (2019) 25, 1185–1193.

[B12] Hirano A. Yumimoto K. Tsunematsu R.. (2013). FBXL21 regulates oscillation of the circadian clock through ubiquitination and stabilization of cryptochromes. Cell 152, 1106–1118. doi: 10.1016/j.cell.2013.01.054 23452856

[B13] Jian S. F. Liao Q. Song H. X. Liu Q. Lepo J. E. Guan C. Y.. (2018). NRT1.1-related NH_4_ ^+^ toxicity is associated with a disturbed 2 balance between NH_4_ ^+^ uptake and assimilation. Plant Physiol. 178, 1473–1488. doi: 10.1104/PP.18.00410 30337453 PMC6288744

[B14] Jin X. C. Guo J. X. Xu Q. J. Hu X. W. Zhang R. J. (2008). Effects of different concentrations of NH_4_ ^+^ on antioxidant system of *Hydrilla verticillata* and *Myriophyllum spicatum* . Ecol. Environ. 17, 1–5. doi: 10.3969/j.issn.1674-5906.2008.01.001

[B15] Kronzucker H. J. Siddiqi M. Y. Glass A. D. M. Britto D. T. (2003). Root ammonium transport effiffifficiency as a determinant in forest colonization patterns: An hypothesis. Physiol. Plant 117, 164–170. doi: 10.1034/j.1399-3054.2003.00032.x

[B16] Lea P. J. Azevedo R. A. (2006). Nitrogen use efficiency. 1. uptake of nitrogen from the soil. Ann. Appl. Biol. 149, 243–247. doi: 10.1111/j.1744-7348.2006.00101.x

[B17] Lea P. J. Miflin B. J. (2003). Glutamate synthase and the synthesis of glutamate in plants. Plant Physiol. Bioch. 41, 555–564. doi: 10.1016/S0981-9428(03)00060-3

[B18] Lei Y.-B. Wang W.-B. Feng Y.-L. Zheng Y.-L. Gong H.-D. (2012). Synergistic interactions of CO_2_ enrichment and nitrogen deposition promote growth and ecophysiological advantages of invading *Eupatorium adenophorum* in southwest China. Planta 236, 1205–1213. doi: 10.1007/s00425-012-1678-y 22684510

[B19] Li C. S. (2013). “Effects of high ammonium stress on seedling growth and its physiological basis in wheat,” (Nanjing (Jiangsu): Nanjing Agricultural University).

[B20] Li Q. Li B. H. Kronzucker H. J. Shi W. M. (2010). Root growth inhibition by NH_4_ ^+^ in *Arabidopsis* is mediated by the root tip and is linked to NH_4_ ^+^ efflux and GMPase activity. Plant Cell Environ. 33, 1529–1542. doi: 10.1111/j.1365-3040.2010.02162.x 20444215

[B21] Li X. P. Tong Y. P. (2007). Physiological and molecular basis of inorganic nitrogen transport in plants. Chin. Bull. Bot. 24, 714–725. doi: 10.3969/j.issn.1674-3466.2007.06.004

[B22] Liu Y. (2017). “Physiological responses of seed germination and seelding growth to elevated ammonium nutrition in winter wheat (*Triticum aestivum* l.),” (Nanjing (Jiangsu): Nanjing Agricultural University).

[B23] Liu Y. H. Zhu Z. J. Wei G. Q. (2004) 40, 680–682. doi: 10.1300/J079v30n03_01

[B24] Li Y. Zhou J. Y. Hao D. L. Yang S. Y. Su Y. H. (2020). *Arabidopsis* under ammonium over-supply: Characteristics of ammonium toxicity in relation to the activity of ammonium transporters. Pedosphere 30, 314–325. doi: 10.1016/S1002-0160(20)60011-X

[B25] Luo J. J. Gao Y. M. Feng W. W. Liu M. C. Qu B. Zhang C.. (2022). Stronger ability to absorb nitrate and associated transporters in the invasive plant *Xanthium strumarium* compared with its native congener. Environ. Exp. Bot. 198, 104851. doi: 10.1016/j.envexpbot.2022.104851

[B26] Miller A. J. Fan X. R. Orsel M. Smith S. J. Wells D. M. (2007). Nitrate transport and signalling. J. Exp. Bot. 58, 2297–2306. doi: 10.1007/978-3-662-44270-8_12 17519352

[B27] Ou X. H. (2018). “Study on the mechanisy and relative alleviation measures of ammonium toxicity to Panax notoginseng,” (Wuhan (Hubei): Huazhong Agricultural University).

[B28] Pignocchi C. Kiddie G. Hernández I. Foster S. J. Asensi A. Taybi T.. (2006). Ascorbate oxidase-dependent changes in the redox state of the apoplast modulate gene transcript accumulation leading to modified hormone signaling and orchestration of defense processes in tobacco. Plant Physiol. 141, 423–435. doi: 10.1104/pp.106.078469 16603663 PMC1475448

[B29] Polesskaya O. G. Kashirina E. I. Alekhina N. D. (2004). Changes in the activity of antioxidant enzymes in wheat leaves and roots as a function of nitrogen source and supply. Russian J. Plant Physiol. 51, 615–620. doi: 10.1023/B:RUPP.0000040746.66725.77

[B30] Shan X. Qin W. B. Zhang Z. C. Yao Y. M. Xiao Y. Dai Z. L. (2018). Effects of low temperature stress on leaf AsA-GSH cycle metabolism in different varieties *Brassica oleracea* l. J. South. Agr. 49, 2230–2235. doi: 10.3969/j.issn.2095-1191.2018.11.17

[B31] Sohlenkamp C. Wood C. C. Roeb G. W. Udvardi M. K. (2003). Characterization of *Arabidopsis* AtAMT2, a high-affinity ammonium transporter of the plasma membrane. Plant Physiol. 130, 1788–1796. doi: 10.1104/pp.008599 PMC166690 12481062

[B32] Sun M. H. (2017). “Study on the nitrogen absorb, transport and molecular mechanism of different nitrogen forms of citrange,” (Changsha (Hunan): Hunan Agricultural University).

[B33] Sun S. M. Chen J. X. Feng W. W. Zhang C. Huang K. Guan M.. (2021). Plant strategies for nitrogen acquisition and their effects on exotic plant invasions. Biodivers. Sci. 29, 72–80. doi: 10.17520/biods.2020072

[B34] Sun C. F. Dai T. B. Cao W. X. (2003). Effect of the enhanced ammonium nutrition on the growth and nitrogen utilization of wheat under different n levels. Plant Nutr. fert. Sci. 9, 33–38+49. doi: 10.3321/j.issn:1008-505X.2003.01.006

[B35] Sun M. H. Lu X. P. Cao X. J. Li J. Xiong J. Xie S. X. (2017). Effect of different forms of nitrogen on the activity of nitrate reductase and expression of the relative genes in citrus sinensis × poncirus trifoliate. J. Fruit Sci. 34, 410–417. doi: 10.13925/j.cnki.gsxb.20160314

[B36] Szczerba M. W. Britto D. T. Balkos K. D. Kronzucker H. J. (2008). Alleviation of rapid, futile ammonium cycling at the plasma membrane by potassium reveals k^+^-sensitive and -insensitive components of NH_4_ ^+^ transport. J. Exp. Bot. 59, 303–313. doi: 10.1093/jxb/erm309 18203690

[B37] Tabuchi M. Abiko T. Yamaya T. (2007). Assimilation of ammonium ions and reutilization of nitrogen in rice (*Oryza sativa* l.). J. Exp. Bot. 58, 2319–2327. doi: 10.1093/jxb/erm016 17350935

[B38] Tegeder M. (2014). Transporters involved in source to sink partitioning of amino acids and ureides: opportunities for crop improvement. J. Exp. Bot. 65, 1865–1878. doi: 10.1093/jxb/eru012 24489071

[B39] Tewari R. K. Kumar P. Shanna P. N. (2007). Oxidative stress and antioxidant responses in young leaves of mulberry plants grown under nitrogen, phosphorus or potassium deficiency. J. Integr. Plant Biol. 49, 313–322. doi: 10.1111/j.1744-7909.2007.00358.x

[B40] Wang Y. Y. Hsu P. K. Tsay Y. F. (2012). Uptake, allocation and signaling of nitrate. Trends Plant Sci. 17, 458–467. doi: 10.1016/j.tplants.2012.04.006 22658680

[B41] Wang P. Li Y. H. Zhang X. M. Li B. G. Yao F. F. (2013) 40, 417–425.

[B42] Wang S. W. Zhu Y. Y. Di T. J. Zeng H. Q. Shen Q. R. Xu G. H. (2009). Effects of ammonium- and nitrate-nutrition on the plasma membrane h^+^-ATPase and proton pump of rice leaves. Plant Nutr. Fert. Sci. 15, 744–749. doi: 10.3321/j.issn:1008-505X.2009.04.002

[B43] Zou N. Qiang X. M. Shi W. M. (2012). Effects of different levels of NH_4_ ^+^ on growth of tomato roots. Soils 44, 827–833. doi: 10.3969/j.issn.0253-9829.2012.05.019

[B44] Zou C. Q. Wang X. F. Zhang F. S. (2004). Preliminary study on the mechanism of ammonium nitrogen inhibiting the growth of sunflower. Plant Nutr. Fert. Sci. 10, 82–85. doi: 10.3321/j.issn:1008-505X.2004.01.016

