# Supplementary material for: Differences and related physiological mechanisms in effects of ammonium on the invasive plant Xanthium strumarium and its native congener X. sibiricum
Source: Front Plant Sci. 2022 Oct 5;13:999748. doi: 10.3389/fpls.2022.999748 (PMC9581188; doi:10.3389/fpls.2022.999748)
Supplement: Supplementary file 1 [file DataSheet_1.docx]

***Supplementary Material***

Table S1 Effects of species (*n* = 2), ammonium nitrogen concentrations (*n* = 8) and their interaction on total biomass, root to shoot ratio and leaf chlorophyll content of *Xanthium strumaricum* and *X. sibiricum* (measured in the first experiment)

Variables and Factors df *F*-value

**Total biomass**

Species (S) 1 **248.875**

Ammonium nitrogen concentration (AN) 7 **6.695**

S × AN 7 **9.848**

**Root to shoot ratio**

Species (S) 1 **81.224**

Ammonium nitrogen concentration (AN) 7 **11.850**

S × AN 7 **3.496**

**Chlorophyll content**

Species (S) 1 **806.872**

Ammonium nitrogen concentration (AN) 7 **7.361**

S × AN 7 **56.686**

Statistically significant effects are highlighted in bold (*P* < 0.05).

Table S2 Effects of species (*n* = 2), ammonium nitrogen concentrations (*n* = 3) and their interaction on variables of *Xanthium strumaricum* and *X. sibiricum* (measured in the second experiment)

*F*-value

Variables and Factors df Leaf Fine root

**Ammonium nitrogen content**

Species (S) 1 **29.501** **40.846**

Ammonium nitrogen concentration (AN) 2 **37.453** **271.725**

S × AN 2 **11.495** **4.510**

**Total nitrogen content**

Species (S) 1 **69.124**  **30.089**

Ammonium nitrogen concentration (AN) 2 **21.258**  **276.169**

S × AN 2 **3.762**  0.583

**Glutamine synthetase activity**

Species (S) 1 **1395.901**  **69.099**

Ammonium nitrogen concentration (AN) 2 0.873 **112.756**

S × AN 2 **9.701**  **32.721**

**Glutamate synthase activity**

Species (S) 1 **2250.77**  **697.596**

Ammonium nitrogen concentration (AN) 2 **423.798**  **30.998**

S × AN 2 **209.907**  **8.599**

**Hydrogen peroxide content**

Species (S) 1 **526.318**  **46.837**

Ammonium nitrogen concentration (AN) 2 **206.776**  **618.769**

S × AN 2 **67.517**  **351.565**

**Malondialdehyde content**

Species (S) 1 **256.512**  **16.513**

Ammonium nitrogen concentration (AN) 2 **207.116**  **431.189**

S × AN 2 **13.841**  **8.095**

**Reduced glutathione content**

Species (S) 1 **104.186**  **17.595**

Ammonium nitrogen concentration (AN) 2 2.154 **3.668**

S × AN 2 **52.769**  **46.438**

**Reduced ascorbic acid content**

Species (S) 1 **20.065**  **35.675**

Ammonium nitrogen concentration (AN) 2 **34.290**  2.003

S × AN 2 **4.882**  **9.844**

Statistically significant effects are highlighted in bold (*P* < 0.05).


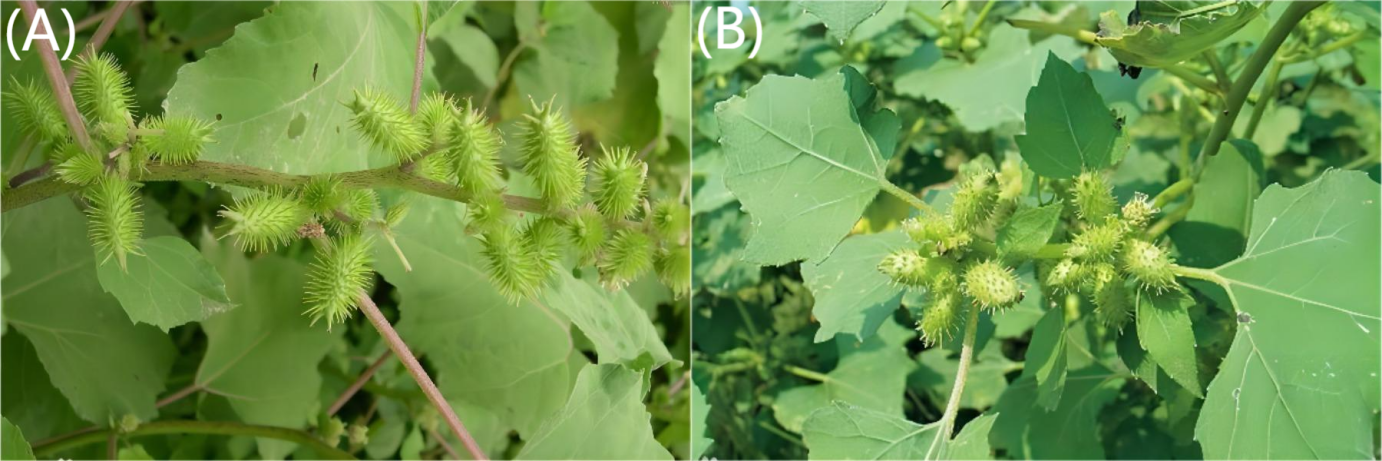


Figure S1 Morphology of the invasive plant *Xanthium strumarium* (A) and its native congener *X. sibiricum* (B) in the field


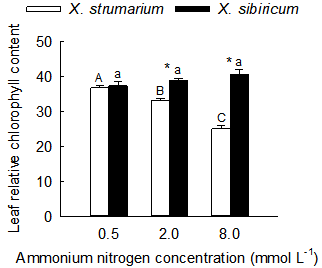


Figure S2 Differences in leaf relative chlorophyll contents between the invasive plant *Xanthium strumaricum* (open bars) and its native congener *X. sibiricum* (closed bars) grown under different ammonium nitrogen levels in the second experiment. The interaction of species and ammonium nitrogen concentration is significant in two-way ANOVA (*P* < 0.05).


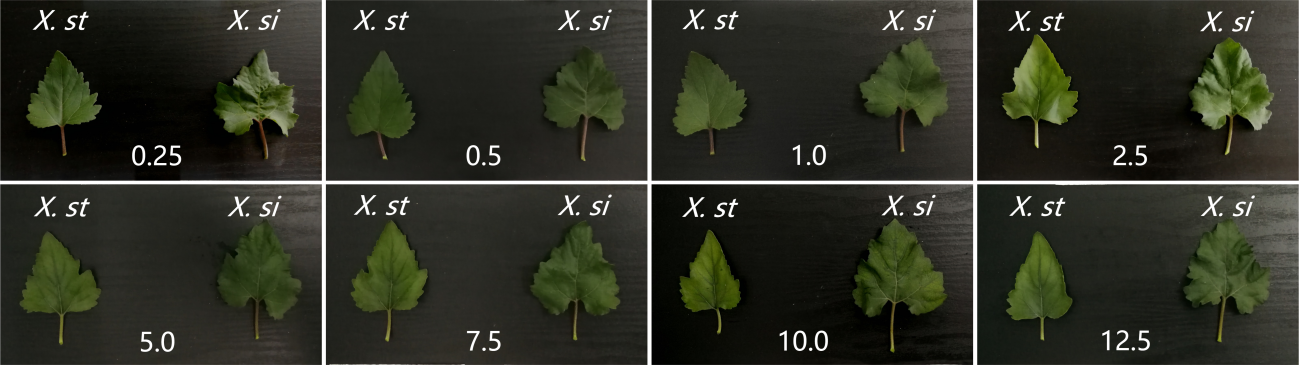


Figure S3 Differences in leaf appearance characteristics (the upper 3^rd^ leaf) between the invasive plant *Xanthium strumaricum* (*X. st*; left in each picture) and its native congener *X. sibiricum* (*X. si*; right in each picture) grown under different concentrations of ammonium nitrogen (indicated by the number on each picture; mmol L^-1^) in the first experiment.


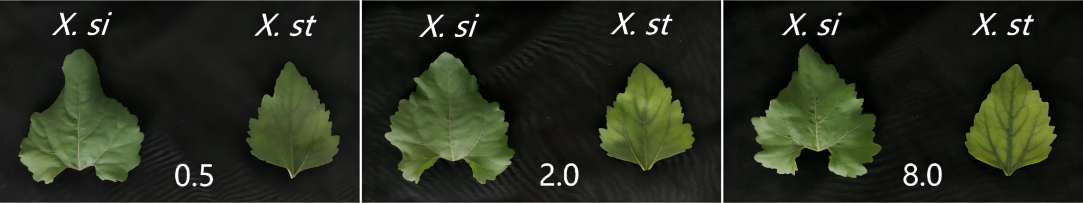


Figure S4 Differences in leaf appearance characteristics (the upper 3^rd^ leaf) between the invasive plant *Xanthium strumaricum* (*X. st*; right in each picture) and its native congener *X. sibiricum* (*X. si*; left in each picture) grown under different concentrations of ammonium nitrogen (indicated by the number on each picture; mmol L^-1^) in the second experiment.
